# Understanding how bacterial collectives organize on surfaces by tracking surfactant flow

**DOI:** 10.1016/j.cossms.2023.101080

**Published:** 2023-04-29

**Authors:** Summer Kasallis, Jean-Louis Bru, Rendell Chang, Quantum Zhuo, Albert Siryaporn

**Affiliations:** a Department of Physics & Astronomy, University of California Irvine, Irvine, CA 92697, USA; b Department of Molecular Biology & Biochemistry, University of California Irvine, Irvine, CA 92697, USA

**Keywords:** Structured optical profilometry, Rhamnolipids, Swarming, *P. aeruginosa*

## Abstract

Swarming is a collective bacterial behavior in which a dense population of bacterial cells moves over a porous surface, resulting in the expansion of the population. This collective behavior can guide bacteria away from potential stressors such as antibiotics and bacterial viruses. However, the mechanisms responsible for the organization of swarms are not understood. Here, we briefly review models that are based on bacterial sensing and fluid mechanics that are proposed to guide swarming in the pathogenic bacterium *Pseudomonas aeruginosa*. To provide further insight into the role of fluid mechanics in *P. aeruginosa* swarms, we track the movement of tendrils and the flow of surfactant using a novel technique that we have developed, Imaging of Reflected Illuminated Structures (IRIS). Our measurements show that tendrils and surfactants form distinct layers that grow in lockstep with each other. The results raise new questions about existing swarming models and the possibility that the flow of surfactants impacts tendril development. These findings emphasize that swarm organization involves an interplay between biological processes and fluid mechanics.

At the single-cell level, bacteria function as individual organisms, lacking any apparent coordination mechanism. However, when their cell density surpasses a critical threshold, bacteria exhibit coordinated behaviors that are reminiscent of processes in multicellular organisms. The bacterial pathogen *Pseudomonas aeruginosa* has two notable collective behaviors when cells reach high density: biofilm formation and swarming motility. These behaviors produce bacterial populations that are profoundly important in natural environments and in the healthcare setting [1–3]. While biofilms and swarms are both dense populations, their cellular states are distinct [3–5]. Individual cells within swarms are highly motile, active, and are associated with low levels of the intracellular nucleotide cyclic di-GMP. In contrast, individual cells in biofilms are less motile, have higher levels of cyclic di-GMP, and adhere to surfaces, to other cells, and to the extracellular matrix. The mechanisms responsible for the structural organization and development of biofilms have been characterized in detail in recent studies [6–9]. However, far less is known about the mechanisms responsible for these aspects of bacterial swarms. Given that swarming is a major bacterial lifestyle, it is important to address the knowledge gap on the mechanisms responsible for this behavior.

Swarming is a form of collective behavior observed in diverse bacteria that grow on porous surfaces such as agar [1]. At the most fundamental level, swarming results in the spatial expansion of a bacterial population. A striking feature of swarms is the diverse patterns that are observed among different species, with some producing dendritic and fractal-like patterns. Significant effort has been devoted to establishing a unified understanding of swarming by collating data and models from multiple organisms. One limitation of this approach is that it potentially convolves the effects of multiple unrelated motility mechanisms. Nuances in motility modalities such as sliding and swimming may be at work in different bacterial organisms and could contribute to the swarming patterns that emerge. For example, the formation of side-by-side cell groups known as rafts are required for swarming in *Proteus mirabilis* and *Bacillus subtilis* [10,11]. These rafts are thought to arise through bundling of multiple flagella with neighboring cells. In contrast, most *P. aeruginosa* strains produce only a single flagellum and are not known to form rafts. Despite the absence of raft formation, swarming is observed in *P. aeruginosa*. To disentangle the effects of rafting and other mechanisms from different species that contribute to swarming, we have focused our discussion on only a single species, *P. aeruginosa*. A significant number of empirical and modeling studies on swarming have been performed on this organism. One of the hallmark features of a *P. aeruginosa* swarm is the appearance of dendritic patterns, also referred to as tendrils, which are dense bacterial growths that extend radially from the center of the population (Fig. 1A). The production of surfactants in the form of rhamnolipids and 3-(3-hydroxyalkanoyloxy)alkanoic acids (HAAs), and flagellar activity are both required for tendril formation [12,13]. Surfactants decrease friction with the surface and flagella propel cells within the swarm. It is unclear how the combination of these mechanisms give rise to tendril formation and swarm expansion. A central question that remains outstanding is what mechanisms are responsible for the tendril patterns that emerge in *P. aeruginosa* swarms?

## 1. Models of tendril development

**Fig. 1.**
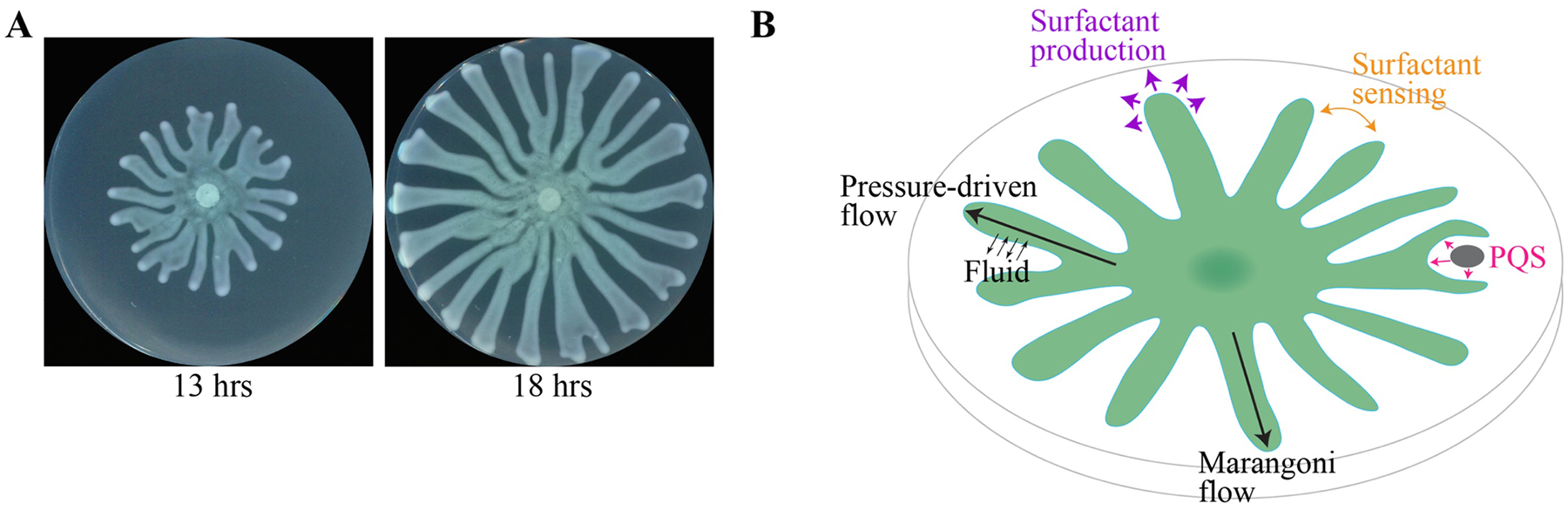
*P. aeruginosa* swarming dynamics and proposed mechanisms that affect swarm organization. (A) Swarms develop tendrils that grow radially from the center. Images of *P. aeruginosa* swarms on 9 cm-diameter Petri dishes after 13 and 18 hrs of growth. (B) Schematic that illustrates existing models of bacterial sensing and fluid mechanics-based processes that contribute to the development and organization of *P. aeruginosa* swarms on agar surfaces. Surfactant produced by the swarm reduces friction between tendrils and the surface. Tendrils avoid other tendrils through surfactant sensing. Tendrils are also repelled by the quorum sensing molecule PQS, which is over-produced by *P. aeruginosa* that are stressed by antibiotics or infected with phage. The expansion of tendrils is facilitated by Marangoni flow and driven by pressure that develops from the influx of fluid from the surface into the tendril.

Multiple models based on bacterial signaling and fluid mechanics propose distinct tendril formation mechanisms (summarized in Fig. 1B). However, the extent to which the models are complementary to or exclusive of one another is unclear. A signaling model proposes that *P. aeruginosa* senses rhamnolipids and HAAs that are secreted by other portions of the swarm [14]. Di-rhamnolipids and HAAs have opposing roles: di-rhamnolipids attract swarming cells and promote tendril formation whereas HAAs are strong repellants and inhibit tendril formation [15,16]. SadB, a signal transduction protein that regulates flagella production, has been proposed to sense rhamnolipids, whether directly or indirectly [14]. In this model, tendril movement is altered through coordination between surfactant production and surfactant sensing. In addition, tendrils are repelled by the quorum sensing molecule 2-heptyl-3-hydroxy-4-quinolone (PQS) [17]. It is possible that PQS is sensed through the PQS-binding quorum sensing regulator PqsR [18] and alters tendril direction in response. While the detection of surfactants and PQS offers a plausible sensing mechanism, this model does not address how tendril direction can be altered in response. The requirement of flagella for tendril formation suggests that swimming is an important process. It is commonly assumed that swimming motility can alter tendril direction, however bacteria cannot swim beyond the edge of tendrils because they are confined by surface tension. It is thus unclear how the action of flagella and swimming motility could alter tendril direction.

Additional insight into tendril formation has come from studies that consider the role of fluid mechanics, in which a swarm is considered to be a thin film of liquid. Their findings suggest that biophysical mechanisms could have a significant role in tendril formation. Two major mechanisms have been proposed to explain how tendrils develop: Marangoni flow and pressure-driven flow [19,20]. Marangoni flow arises from a surface tension gradient that is created by a decrease in rhamnolipid production from the center of the swarm towards the leading edge of tendril tips [19]. This creates a flow of liquid within the tendril that drives the tendril towards increasing surface tension. In the pressure-driven flow model, an influx of water from the agar layer into the swarm layer creates a pressure that drives the outward expansion of the tendril [20]. Several models based on swarming in *Escherichia coli* suggest that osmolytes produced by bacteria are responsible for driving the influx of water into the swarm layer [21–23]. A notable feature of both these fluid mechanics-based models is that tendril formation does not require the bacterial detection of rhamnolipids or other molecules, in contrast to the sensing models discussed above. Bacterial sensing, Marangoni flow, and pressure-driven flow may all work in parallel to form tendrils (Fig. 1B), though the relative contribution of each mechanism is unknown. Thus, there is a need for further evaluation of these models using experimental methods.

A significant challenge in validating the thin liquid film interpretation of swarming is tracking the surfactant component of a swarm, given that surfactants produced by *P. aeruginosa* are optically transparent. A number of techniques can capture distinct aspects of swarms. For example, the bulk of swarms can be imaged at the cm-scale using widely-available consumer cameras or scanners, though these methods do not capture the surfactant [24,25]. Individual cell trajectories within swarms can be captured using brightfield and fluorescence microscopy combined with the use of time-lapse imaging and computational analysis [26]. Movements within the swarm can also be tracked using microbubbles of a water-insoluble surfactant, MgO particles, and gold nanorods [27–29]. Characterization of surfactants has been performed using methylene blue and Nile red dyes [30,31] as well as drop collapse assays [32]. However, these approaches can perturb swarms. Dyes can alter surfactant and swarm dynamics while drop collapse assays require removal of surfactant from the swarm.

## 2. Visualizing the dynamics of the surfactant layer through IRIS

To better measure the dynamics of surfactants and swarms without perturbing them, our lab developed an imaging method that relies on surface reflection. Here, the swarm surface is illuminated by a structured image and the reflection off the surface is captured by a camera. The structured image consists of alternating black and white squares which increase the contrast of surface features, including the liquid–air interface at the edge of the swarm. This technique, which we refer to as Imaging of Reflected Illuminated Structures (IRIS), provides high resolution images of an optically transparent liquid. The technique is noninvasive, requires no modification of the swarm or growth conditions, and is simple and cost-effective.

**Fig. 2.**
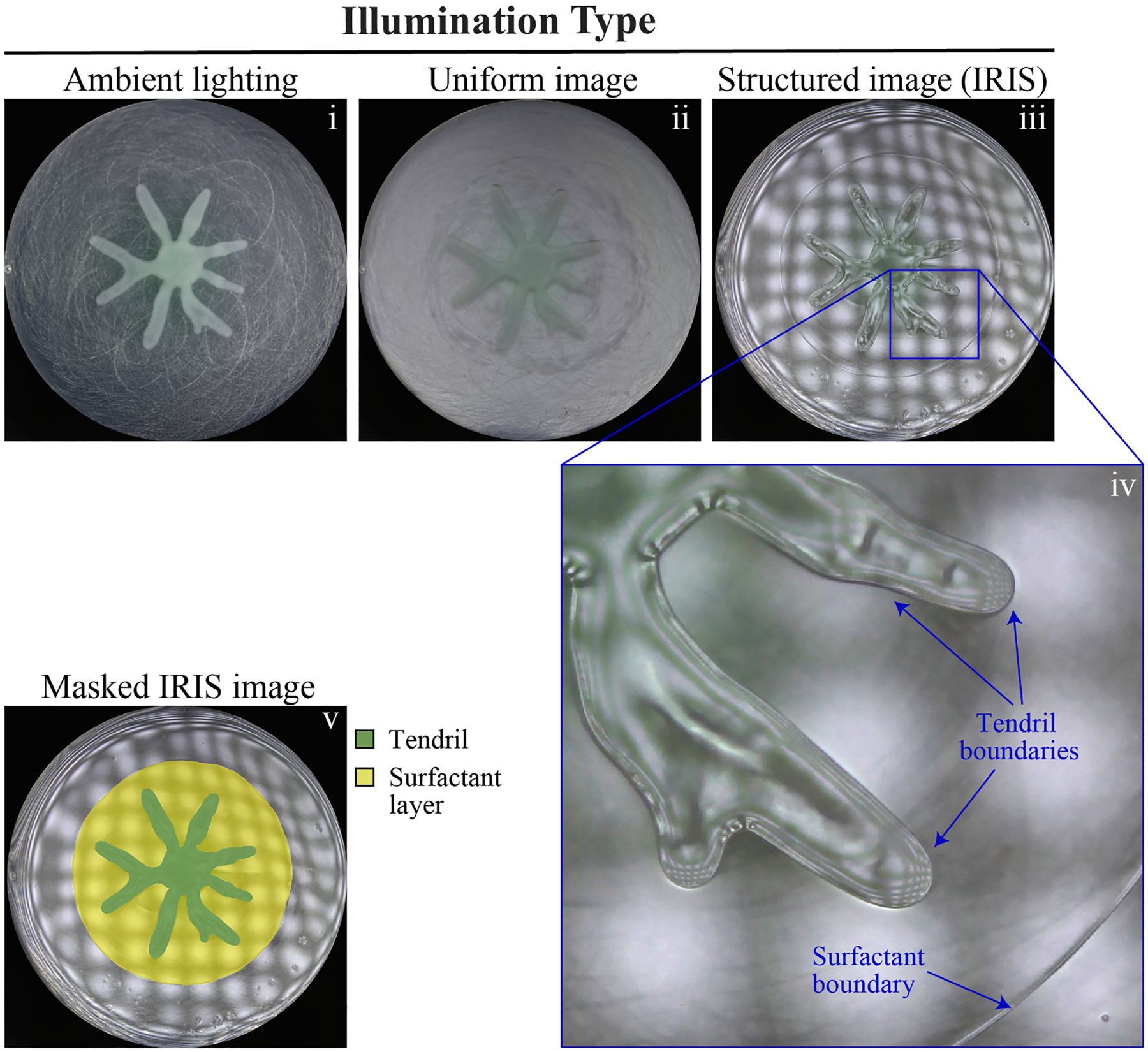
IRIS imaging non-invasively reveals tendril and surfactant boundaries. Illumination of swarming plates using: (i) ambient light, (ii) a reflected light source consisting of a uniform image, and (iii) a reflected light source consisting of a structured image, which produces the IRIS image. (iv) Magnification of a section of the IRIS image. (v) False-colored mask of the IRIS image, indicating tendrils (dark green) and surfactant (yellow).

We imaged a *P. aeruginosa* swarm using multiple methods of illumination. Under ambient lighting, in which illumination was provided by ceiling lights of the lab, tendrils were observed but surfactant was not visible (Fig. 2 i). A similar result was observed using an illumination setup in which a single uniform white image was reflected off of the swarm surface (Fig. 2 ii). When the illumination was changed from a uniform image to one that was structured in the form of repeating black and white squares, additional surface details were observed. In particular, tendril topography and a layer of surfactant that surrounded the swarm tendrils were visible (Fig. 2 iii, iv). This method relies on the effect of the contrast created by the structured image. For example, a perfectly flat surface produces an undistorted reflection of the structured image. On the other hand, changes in curvature that are present on a non-flat surface produce distortions in the reflected structured image. These distortions bring non-flat surface features into the foreground of the image. Liquid boundaries and swarming tendrils, which have large changes in surface curvature, appear as distortions of the structured image.

Imaging of *P. aeruginosa* swarms using IRIS revealed new details that can better address a fluid mechanics-based interpretation of swarming. Two well-defined boundaries were observed: a boundary around the outer edge of the tendrils that encompassed the bacterial growth, and a larger circular boundary that encompassed all the tendrils (Fig. 2 iii, iv). The larger boundary is consistent with previous observations of surfactants that are produced by cells in the tendrils [31,33]. In the IRIS image, surfactants appeared to form a layer on which the tendrils formed an additional layer (Fig. 2 iv). Using edge detection software, masks were overlaid on the image to demarcate the tendrils and surfactant (Fig. 2 v).

**Fig. 3.**
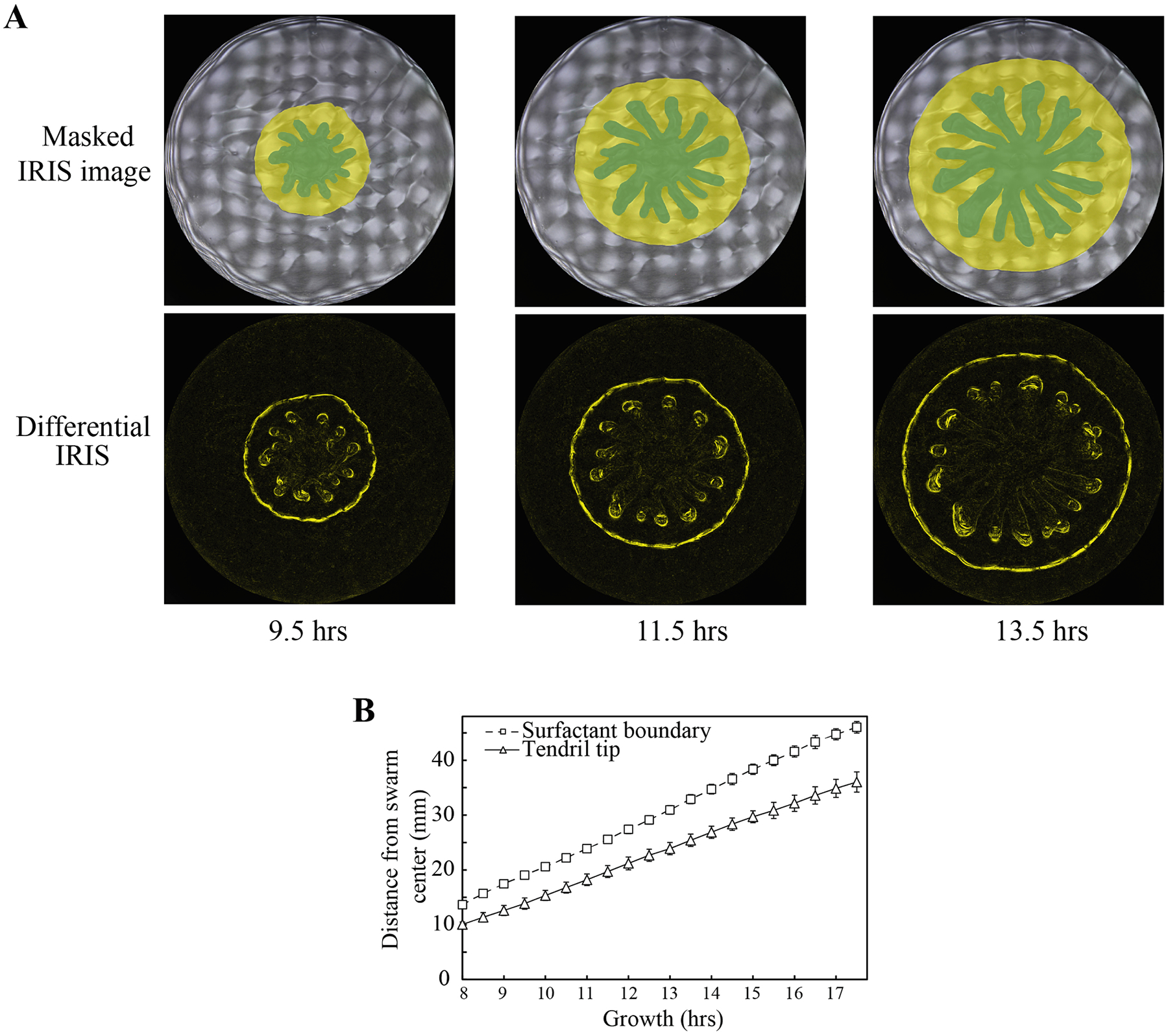
Differential IRIS imaging reveals coupling between the growth of the tendril tips and surfactant boundary. (A) Masked IRIS and differential IRIS images of swarms at 9.5, 11.5, and 13.5 hrs of growth. Masked IRIS images indicate tendrils (dark green) and surfactant (yellow). Differential images indicate movement (yellow) within respective masked IRIS images during a 5-minute interval. (B) Distance of tendril tips and surfactant boundary from the center of the swarming plate. Distances were measured for all tendril tips and at 8 different uniformly distributed positions on the surfactant boundary. Data points represent average distances and error bars indicate standard deviation.

To investigate the dynamics of the tendril and surfactant layers, we performed time-lapse imaging of the swarm using IRIS. By performing an image subtraction between successive images, we identified portions of the swarm that have non-zero instantaneous speeds (Fig. 3A). This differential IRIS approach identifies parts of the swarm that are actively expanding or moving. The greatest movements within the swarm were observed at the tendril tips where the tendrils meet the surfactant layer, and at the outermost boundary of the surfactant layer (Fig. 3A). To our surprise, the tendril tips and the surfactant boundary expanded simultaneously, with a strong correlation (r = 0.99) between their rates of expansion (Fig. 3B). This result suggests that the tendril and surfactant layers are coupled during swarm expansion.

These observations provide new insight into the structure of the swarm and suggest that swarms consist of multiple distinct layers rather than a single thin film of liquid. Additionally, the coupling observed between the tendril and surfactant layers raises the possibility that alterations in one layer could impact the movement of the other. For example, changes in the flow of the surfactant layer could alter the movement of tendrils. Such a mechanism would have significant implications on how tendrils develop. In upcoming work from our lab, it is reported that *P. aeruginosa* tendrils are deflected by molecules that alter the flow of the surfactant layer [34]. Using IRIS, it is found that a range of molecules including PQS and those produced by the bacterium *Staphylococcus aureus* alter the flow of the surfactant layer and concomitantly, the movement of the tendrils. The data suggest that surfactant flow can guide tendril movement and have an important role in swarm organization. Many bacterial species found in natural and healthcare settings produce surfactants (i.e., surfactin from *B. subtilis* and rhamnolipids from *P. aeruginosa*), but how surfactant flow among these species is affected by environmental factors has not been explored. Such interactions could have large impacts on the spatial organization of swarms.

IRIS has many advantages and potential applications. It is highly effective at probing surface topography, may be applied to a broad range of materials, and is relatively simple and cost-effective compared to conventional methods of optical profilometry. Our application of IRIS highlights the need to consider the role of fluid mechanics-based processes in swarming. Tracking surfactant flows within swarms will expand the knowledge of how swarms develop and organize. At the most fundamental level, swarming is a mechanism that results in volumetric expansion of a bacterial population on porous surfaces, which are materials that are ubiquitous in natural and healthcare-related settings. Understanding swarming mechanisms will thus provide insights into how bacterial population expansion occurs at the fundamental level.

## 3. Methods

### 3.1. Growth conditions

*P. aeruginosa* strain AFS27E.1 [17], which is derived from strain PA14, was streaked onto LB Broth, Miller (BD, Franklin Lakes, NJ) containing 2% Bacto Agar (BD, Franklin Lakes, NJ). Single colonies were inoculated into LB Broth and cultured in a roller drum at 17.5 rpm at 37 °C for 16 to 18 hrs. Swarms were cultured by spotting 5 μL of culture on swarming Petri dishes, as described in [17,25] and incubated at 37 °C at a humidity of 47%. Swarms in Fig. 1 were imaged using an Epson scanner as described in [17,25].

### 3.2. IRIS imaging

Polystyrene Petri dish surfaces were treated with a metal wire brush before swarming medium was added. Dishes containing swarming medium and bacterial strains were illuminated directly above by a 27” ASUS LCD monitor with 1920×1080 resolution (Model VE278Q, ASUS, Taipei, Taiwan) that displayed an image containing 31 × 17 (horizontal × vertical) alternating black and white squares. Images were acquired using a Canon EOS Rebel T7 DSLR camera with 18–55 mm f/3.5–5.6 lens (Model 2727C002, Canon, Huntington, NY) that was angled 16° away from a vertical line directly above the dish. The lid of the Petri dish was removed prior to image acquisition using a robotic arm. Masks of the IRIS images were constructed using the Magnetic Lasso tool in Adobe Photoshop (Adobe, San Jose, California). Differential IRIS images were constructed using the imabsdiff function in Matlab R2017a (Mathworks, Natick, MA).

## Data Availability

Data will be made available on request.
